# Supplementary material for: Discriminative stimuli are sufficient for incubation of cocaine craving
Source: eLife. 2019 Feb 25;8:e44427. doi: 10.7554/eLife.44427 (PMC6417857; doi:10.7554/eLife.44427)
Supplement: Figure 1—source data 1. [file elife-44427-fig1-data1.docx]

## Figure 1-source data 1. Statistical output for Experiment 1: Incubation of discriminative stimulus-controlled cocaine seeking (analyses pertaining to Figure 1 are highlighted in grey)

| **Experimental phase** | **Behavioral measure** | **Factors in analysis** | **F-value** | **P-value** | **Figure** |
| --- | --- | --- | --- | --- | --- |
| Self-administration | Infusions | Session x Sex |  |  |  |
|  |  | Session | F_5,60_=1.84 | 0.1192 |  |
|  |  | Sex | F_1,12_=0.12 | 0.7307 |  |
|  |  | Session x Sex | F_5,60_=0.55 | 0.7397 |  |
|  | Presses | Session x Sex |  |  |  |
|  |  | Session | F_5,60_=0.66 | 0.6555 |  |
|  |  | Sex | F_1,12_=0.77 | 0.3976 |  |
|  |  | Session x Sex | F_5,60_=0.28 | 0.9244 |  |
|  | Infusions | Session (collapsed across Sex) |  |  | 1C |
|  |  | Session | F_5,65_=1.90 | 0.1061 |  |
|  | Presses | Session (collapsed across Sex) |  |  | 1C |
|  |  | Session | F_5,65_=0.69 | 0.6299 |  |
| Discrimination training | Trials | DS x Session x Sex |  |  |  |
|  |  | DS | F_1,12_=992.35 | <0.0001 |  |
|  |  | Session | F_9,108_=1.55 | 0.1407 |  |
|  |  | Sex | F_1,12_=0.30 | 0.5962 |  |
|  |  | DS x Sex | F_1,12_=2.68 | 0.1276 |  |
|  |  | DS x Session | F_9,108_=1.72 | 0.0926 |  |
|  |  | Session x Sex | F_9,108_=1.05 | 0.4047 |  |
|  |  | DS x Session x Sex | F_9,108_=2.16 | 0.0302 |  |
|  | Presses | DS x Session x Sex |  |  |  |
|  |  | DS | F_1,12_=178.08 | <0.0001 |  |
|  |  | Session | F_9,108_=1.47 | 0.1684 |  |
|  |  | Sex | F_1,12_=1.81 | 0.2038 |  |
|  |  | DS x Sex | F_1,12_=3.89 | 0.0720 |  |
|  |  | DS x Session | F_9,108_=2.89 | 0.0042 |  |
|  |  | Session x Sex | F_9,108_=1.35 | 0.2225 |  |
|  |  | DS x Session x Sex | F_9,108_=2.79 | 0.0056 |  |
|  | Trials | DS x Session (collapsed across Sex) |  |  | 1C |
|  |  | DS | F_1,13_=948.21 | <0.0001 |  |
|  |  | Session | F_9,117_=1.48 | 0.1645 |  |
|  |  | DS x Session | F_9,117_=1.65 | 0.1104 |  |
|  | Presses | DS x Session (collapsed across Sex) |  |  | 1C |
|  |  | DS | F_1,13_=161.63 | <0.0001 |  |
|  |  | Session | F_9,117_=1.39 | 0.2014 |  |
|  |  | DS x Session | F_9,117_=2.62 | 0.0085 |  |

| **Experimental phase** | **Behavioral measure** | **Factors in analysis** | **F-value** | **P-value** | **Figure** |
| --- | --- | --- | --- | --- | --- |
| Relapse test | Trials | DS x Day x Sex |  |  |  |
|  |  | DS | F_1,12_=270.55 | <0.0001 |  |
|  |  | Day | F_6,66_=11.25 | <0.0001 |  |
|  |  | Sex | F_1,12_=9.92 | 0.0084 |  |
|  |  | DS x Sex | F_1,12_=12.99 | 0.0036 |  |
|  |  | DS x Day | F_6,66_=4.82 | 0.0004 |  |
|  |  | Day x Sex | F_6,66_=2.02 | 0.0752 |  |
|  |  | DS x Day x Sex | F_6,66_=0.76 | 0.6020 |  |
|  | Presses | DS x Day x Sex |  |  |  |
|  |  | DS | F_1,12_=172.77 | <0.0001 |  |
|  |  | Day | F_6,66_=9.15 | <0.0001 |  |
|  |  | Sex | F_1,12_=3.51 | 0.0854 |  |
|  |  | DS x Sex | F_1,12_=3.22 | 0.0981 |  |
|  |  | DS x Day | F_6,66_=7.74 | <0.0001 |  |
|  |  | Day x Sex | F_6,66_=0.83 | 0.5529 |  |
|  |  | DS x Day x Sex | F_6,66_=0.28 | 0.9440 |  |
|  | Trials | DS x Day (collapsed across Sex) |  |  | 1D |
|  |  | DS | F_1,13_=257.53 | <0.0001 |  |
|  |  | Day | F_6,72_=9.68 | <0.0001 |  |
|  |  | DS x Day | F_6,72_=4.30 | 0.0009 |  |
|  | Presses | DS x Day (collapsed across Sex) |  |  | 1D |
|  |  | DS | F_1,13_=182.25 | <0.0001 |  |
|  |  | Day | F_6,72_=8.94 | <0.0001 |  |
|  |  | DS x Day | F_6,72_=7.95 | <0.0001 |  |
| Reinstatement test | Trials | DS x Treatment x Sex |  |  |  |
|  |  | DS | F_1,9_=101.26 | <0.0001 |  |
|  |  | Treatment | F_3,27_=11.37 | <0.0001 |  |
|  |  | Sex | F_1,9_=4.57 | 0.0613 |  |
|  |  | DS x Sex | F_1,9_=0.09 | 0.7703 |  |
|  |  | DS x Treatment | F_3,27_=11.89 | <0.0001 |  |
|  |  | Treatment x Sex | F_3,27_=0.73 | 0.5409 |  |
|  |  | DS x Treatment x Sex | F_3,27_=0.23 | 0.8718 |  |
|  | Presses | DS x Treatment x Sex |  |  |  |
|  |  | DS | F_1,9_=41.43 | 0.0001 |  |
|  |  | Treatment | F_3,27_=7.89 | 0.0006 |  |
|  |  | Sex | F_1,9_=1.82 | 0.2099 |  |
|  |  | DS x Sex | F_1,9_=0.53 | 0.4868 |  |
|  |  | DS x Treatment | F_3,27_=8.64 | 0.0003 |  |
|  |  | Treatment x Sex | F_3,27_=0.63 | 0.6029 |  |
|  |  | DS x Treatment x Sex | F_3,27_=0.26 | 0.8560 |  |
|  | Trials | DS x Treatment (collapsed across Sex) |  |  | 1E |
|  |  | DS | F_1,10_=108.66 | <0.0001 |  |
|  |  | Treatment | F_3,30_=15.35 | <0.0001 |  |
|  |  | DS x Treatment | F_3,30_=12.42 | <0.0001 |  |
|  | Presses | DS x Treatment (collapsed across Sex) |  |  | 1E |
|  |  | DS | F_1,10_=45.73 | <0.0001 |  |
|  |  | Treatment | F_3,30_=8.31 | 0.0004 |  |
|  |  | DS x Treatment | F_3,30_=9.45 | 0.0001 |  |
