# Supplementary material for: Discriminative stimuli are sufficient for incubation of cocaine craving
Source: eLife. 2019 Feb 25;8:e44427. doi: 10.7554/eLife.44427 (PMC6417857; doi:10.7554/eLife.44427)
Supplement: Figure 2—source data 1. [file elife-44427-fig2-data1.docx]

## Figure 2-source data 1. Statistical output for Experiment 2: Abatement of discriminative stimulus-controlled palatable food-seeking (analyses pertaining to Figure 2 are highlighted in grey)

| **Experimental phase** | **Behavioral measure** | **Factors in analysis** | **F-value** | **P-value** | **Figure** |
| --- | --- | --- | --- | --- | --- |
| Self-administration | Pellets | Session x Sex |  |  |  |
|  |  | Session | F_2,28_=5.57 | 0.0092 |  |
|  |  | Sex | F_1,14_=0.89 | 0.3620 |  |
|  |  | Session x Sex | F_2,28_=0.39 | 0.6831 |  |
|  | Presses | Session x Sex |  |  |  |
|  |  | Session | F_2,28_=6.98 | 0.0035 |  |
|  |  | Sex | F_1,14_=0.05 | 0.8317 |  |
|  |  | Session x Sex | F_2,28_=0.11 | 0.8989 |  |
|  | Pellets | Session (collapsed across Sex) |  |  | 2C |
|  |  | Session | F_2,30_=5.75 | 0.0077 |  |
|  | Presses | Session (collapsed across Sex) |  |  | 2C |
|  |  | Session | F_2,30_=7.44 | 0.0024 |  |
| Discrimination training | Trials | DS x Session x Sex |  |  |  |
|  |  | DS | F_1,13_=998.32 | <0.0001 |  |
|  |  | Session | F_10,130_=15.36 | <0.0001 |  |
|  |  | Sex | F_1,13_=1.78 | 0.2053 |  |
|  |  | DS x Sex | F_1,13_=1.38 | 0.2618 |  |
|  |  | DS x Session | F_10,130_=8.06 | <0.0001 |  |
|  |  | Session x Sex | F_10,130_=0.99 | 0.4539 |  |
|  |  | DS x Session x Sex | F_10,130_=0.65 | 0.7660 |  |
|  | Presses | DS x Session x Sex |  |  |  |
|  |  | DS | F_1,13_=571.09 | <0.0001 |  |
|  |  | Session | F_10,130_=15.05 | <0.0001 |  |
|  |  | Sex | F_1,13_=1.43 | 0.2537 |  |
|  |  | DS x Sex | F_1,13_=5.89 | 0.0304 |  |
|  |  | DS x Session | F_10,130_=2.34 | 0.0141 |  |
|  |  | Session x Sex | F_10,130_=0.78 | 0.6461 |  |
|  |  | DS x Session x Sex | F_10,130_=0.59 | 0.8216 |  |
|  | Trials | DS x Session (collapsed across Sex) |  |  | 2C |
|  |  | DS | F_1,14_=577.71 | <0.0001 |  |
|  |  | Session | F_10,140_=15.63 | <0.0001 |  |
|  |  | DS x Session | F_10,140_=2.31 | 0.0151 |  |
|  | Presses | DS x Session (collapsed across Sex) |  |  | 2C |
|  |  | DS | F_1,14_=1014.94 | <0.0001 |  |
|  |  | Session | F_10,140_=15.42 | <0.0001 |  |
|  |  | DS x Session | F_10,140_=8.22 | <0.0001 |  |

| **Experimental phase** | **Behavioral measure** | **Factors in analysis** | **F-value** | **P-value** | **Figure** |
| --- | --- | --- | --- | --- | --- |
| Relapse test | Trials | DS x Day x Sex |  |  |  |
|  |  | DS | F_1,13_=124.95 | <0.0001 |  |
|  |  | Day | F_4,52_=7.43 | <0.0001 |  |
|  |  | Sex | F_1,13_=6.56 | 0.0237 |  |
|  |  | DS x Sex | F_1,13_=0.02 | 0.8961 |  |
|  |  | DS x Day | F_4,52_=13.12 | <0.0001 |  |
|  |  | Day x Sex | F_4,52_=4.11 | 0.0058 |  |
|  |  | DS x Day x Sex | F_4,52_=0.43 | 0.7849 |  |
|  | Presses | DS x Day x Sex |  |  |  |
|  |  | DS | F_1,13_=107.56 | <0.0001 |  |
|  |  | Day | F_4,52_=8.68 | <0.0001 |  |
|  |  | Sex | F_1,13_=0.01 | 0.9134 |  |
|  |  | DS x Sex | F_1,13_=3.33 | 0.0910 |  |
|  |  | DS x Day | F_4,52_=18.11 | <0.0001 |  |
|  |  | Day x Sex | F_4,52_=2.15 | 0.0875 |  |
|  |  | DS x Day x Sex | F_4,52_=1.91 | 0.1233 |  |
|  | Trials | DS x Day (collapsed across Sex) |  |  | 2D |
|  |  | DS | F_1,14_=133.04 | <0.0001 |  |
|  |  | Day | F_4,56_=5.57 | 0.0008 |  |
|  |  | DS x Day | F_4,56_=14.29 | <0.0001 |  |
|  | Presses | DS x Day (collapsed across Sex) |  |  | 2D |
|  |  | DS | F_1,14_=101.92 | <0.0001 |  |
|  |  | Day | F_4,56_=8.57 | <0.0001 |  |
|  |  | DS x Day | F_4,56_=17.44 | <0.0001 |  |
